# The Correlation between the Use of the Proton Pump Inhibitor and the Clinical Efficacy of Immune Checkpoint Inhibitors in Non-Small Cell Lung Cancer

**DOI:** 10.1155/2022/1001796

**Published:** 2022-07-09

**Authors:** Da-Hai Hu, Wan-Ching Wong, Jia-Xin Zhou, Ji Luo, Song-Wang Cai, Hong Zhou, Hui Tang

**Affiliations:** ^1^Department of General Surgery, The First Affiliated Hospital of Jinan University, Guangzhou 510632, China; ^2^International School, Jinan University, Guangzhou, Guangdong 510632, China; ^3^Department of Thoracic Surgery, The First Affiliated Hospital of Jinan University, Guangzhou 510632, China; ^4^Department of Obstetrics and Gynecology, The First Affiliated Hospital of Jinan University, Guangzhou 510632, China; ^5^Clinical Medicine Research Institute, The First Affiliated Hospital of Jinan University, Guangzhou 510632, China; ^6^Jiangmen Maternity and Child Health Care Hospital, Huizhou 52900, China

## Abstract

**Background:**

To determine if the use of the Proton Pump Inhibitors (PPI) impacts the clinical efficacy of Immune Checkpoint Inhibitors (ICIs) in Non-Small Cell Lung Cancer (NSCLC), a meta-analysis was conducted.

**Method:**

Eleven studies from PubMed, EMBASE, Cochrane Library, Web of Science, and other databases up to May 2022, were selected. The pertinent clinical outcomes were assessed by applying the Progression-free survival (PFS), Overall Survival (OS), Hazard Ratio (HR), and 95% Confidence Interval (CI).

**Result:**

This study included eleven articles containing 7,893 NSCLC patients. The result indicated that PPI use was dramatically related to poor OS (HR: 1.30 [1.10–1.54]), and poor PFS (HR: 1.25 [1.09–1.42]) in case of patients treated with ICIs. With regard to the subgroup analysis, PPI use was dramatically associated with poor OS (Europe: HR = 1.48 [1.26, 1.74], Worldwide: HR = 1.54 [1.24, 1.91]), and poor PFS (Europe: HR = 1.36 [1.18, 1.57], Worldwide: HR = 1.34 [1.16, 1.55]) in patients from Europe and multi-center studies across the world, poor OS in patients with age less than or equal to 65 (HR = 1.56 [1.14, 2.15]), poor PFS in patients aged more than 65 (HR = 1.36 [1.18, 1.57]), poor OS for patients receiving with PD-1 (HR = 1.37 [1.04, 1.79]), poor PFS for patients receiving with PD-L1 (HR = 1.33 [1.19, 1.49]), and poor OS (−30: HR = 1.89 [1.29, 2.78], ±30: HR = 1.44 [1.27, 1.64]) and poor PFS (−30: HR = 1.51 [1.11, 2.05], ±30: HR = 1.32 [1.20, 1.45]) for patients who received PPI at 30 days before and/or after starting the ICIs treatment.

**Conclusion:**

Our meta-analysis indicated that PPI combined with ICIs in the treatment of NSCLC patients could result in poor OS and PFS. PPI use should be extremely cautious in clinical practices to avoid the impact on the efficacy of the ICIs.

## 1. Introduction

Non-small cell lung cancer (NSCLC) is one of the most common cancers, accounting for about 80% of all lung cancers. According to incomplete statistics, NSCLC kills 1.6 million people worldwide each year [1]. The main pathologic types of NSCLC include squamous cell carcinoma, adenocarcinoma, and large cell carcinoma [2]. Compared with small cell carcinoma, the growth and division rate of cancer cells in NSCLC is slow, and the diffusion and metastasis occur relatively late. However, after systematic treatment, the 5-year survival rate of some NSCLC patients is still not ideal [3]. Nowadays, for NSCLC, a single treatment plan, such as surgery, radiotherapy, or immunotherapy, may be difficult to achieve ideal results. Therefore, combination therapy has gradually come into people's vision, such as chemotherapy or radiotherapy after surgery, and Proton Pump Inhibitor (PPI) combined with Immune Checkpoint Inhibitors (ICIs). However, the clinical efficacy of these combined therapies for patients is still unclear, and more relevant studies are needed to explore.

As an effective inhibitor of gastric acid secretion, the PPI has been used widely to treat the hypersecretion of gastric acid and other related diseases around the world, for instance, Gastroesophageal Reflux Disease (GERD) and gastric ulcers [4]. Studies have indicated that use of PPI for a long time could increase the risk of related tissue histopathological changes, and could lead to the disorder of normal colonies in the gastrointestinal tract, greatly increasing the risk of gastrointestinal infection [5–7]. In recent years, since PPI could enhance the sensitivity of cancer patients towards chemotherapy, hence, it has gained prominence in the field of tumor treatment [8, 9]. Nevertheless, the efficacy and risk of the use of PPI are different for various cancer types [10]. However, the efficacy of the use of PPI in the treatment of cancer is not very clear, and needs further research.

Presently, the cancer immunotherapy mainly includes the following three kinds, ICIs and adoptive cell therapy, operating the immunologic defense to differentiate, and attack tumor cells [11]. Among them, the ICIs have been used in a widespread manner in the neighborhood of tumor treatment, greatly improving the strategy of treating related cancer. Cytotoxic drug T lymphocyte associated antigen 4 (CTLA-4) inhibitor, programmed cell death ligand 1 (PD-L1), as well as programmed cell death 1 (PD-1) are the three kinds of ICIs widely used clinically [12, 13]. However, certain controversial aspects of cancer immunotherapy still remain. As the immune system could be over activated during immunotherapy, bring with it, it could bring serious side effects to the patients, and the adverse reactions in individual cases were serious and even life-threatening at times [14]. These illustrated that the clinical efficacy of ICIs was not very clear. Today, with the popularity of the combination therapy, the ICIs are often combined with the PPI. In one study, for NSCLC patients, the PPI combined with ICIs led to a negative result, nevertheless, in case of the melanoma patients, it produced a positive result [15]. Hence, it is controversial whether the clinical efficacy of ICIs in NSCLC is related to the use of the PPI.

This study was intended to determine if there was any correlation between the clinical efficacy of the ICIs in NSCLC and the use of PPI.

## 2. Materials and Method

### 2.1. Search Strategy

**Figure 1:**
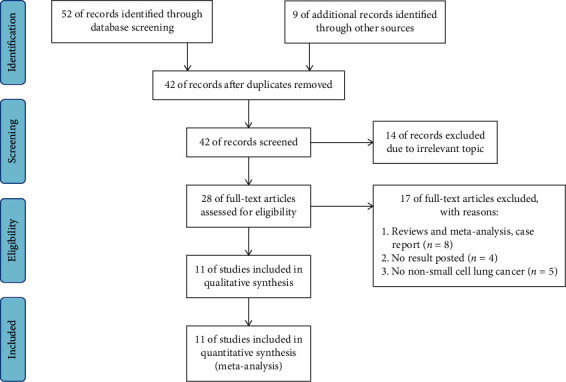
The flow chart of study selection.

The literatures involved in this study were independently screened by two researchers (D. H. and W. W.) to determine whether they met the inclusion or exclusion criteria, and any differences would be resolved by consensus with third party researcher (J. Z.). Our search strategy is as illustrated in Figure 1, searching the studies from PubMed, EMBASE, Cochrane Library, and Web of Science databases up to May 2022. The keywords searched were, “Non-small Cell Lung Cancer,” “Non-Small Cell Lung Cancer,” “carcinoma, non-small-cell lung” “Non-Small Cell Lung Carcinoma,” “Lung Carcinoma, Non-Small-Cell,” “programmed death-ligand 1 inhibitor,” “PD-L1 inhibitor,” “Immunotherapy,” “programmed death receptor 1 inhibitor,” “PD-1 inhibitor,” “cytotoxic T lymphocyte antigen-4 inhibitor,” “CTLA-4 inhibitor,” and “proton pump inhibitor.”

### 2.2. The Criteria for Inclusion and Exclusion

The criteria for inclusion were: (1) The collected literature involving the usage of PPI and the clinical efficacy in NSCLC of the ICIs; (2) Use of PPI done before and/or after starting the ICIs treatment; (3) Patients received just the ICIs treatments or combined with PPI; (4) The inclusion of the non-using PPI and using PPI groups; and (5) The outcome of study should contain the Overall Survival (OS) and/or Progression-Free Survival (PFS), Hazard Ratio (HR), and 95% Confidence Intervals (CIs). The exclusion criteria were as under: (1) Repetitive studies; (2) Non-human studies; (3) The study report was not in English; and (4) The reviews and meta-analyses, or the case report.

### 2.3. Data Extracting/

The result information like 95% CI of OS and/or PFS, HR, duration of exposure to PPI, cancer type, PPI treatment, type of ICIs treatment, sample size, age, region, first author, and year of publication were extracted from the studies that were included. To reduce the influence of the confounding factors, the multivariate analysis was selected to calculate the HRs value to the extent possible.

### 2.4. Quality Assessment

The literature included in the study were retrospective studies and the quality evaluation of research referred to the Newcastle—Ottawa Quality Assessment Scale (NOS) [16]. The evaluation was scored with respect to three aspects, selection of topic, comparability, and evaluation of results. For the NOS system, a score of 6 or more of studies was defined as high quality [17].

### 2.5. Statistical Analysis

The HR and 95% CI of OS and/or PFS were meta-analyzed by applying the Review Manager 5.4 software for Win, while HR >1.0 was considered as poor OS or poor PFS in the outcomes. The funnel plot assessed the publication bias. The heterogeneity of the studies included was assessed by *I*^2^ statistics, and the sensitivity analysis, Begg's tests, and Egger's tests of studies were evaluated by Stata 15 software for Win. When *I*^2^ was greater than 50%, it was regarded that the research had great heterogeneity, and the random effect model was adopted. The extracted data were analyzed by the dichotomous, Mantel–Haenszel method model. In this study, *P* values <0.05 were considered as statistically significant.

## 3. Result

### 3.1. Selection of Study

Figure 1 illustrates the flow chart of the selection of studies. 52 studies that were searched from the database were included in this study, with 9 supplemental studies from other databases. After repetitive studies (*n* = 19) and studies of unrelated topics (*n* = 14) were deleted, with 42 articles being selected. Subsequently, according to the above including and excluding criteria, 17 articles were excluded, including reviews, and meta-analysis, case report (*n* = 8), no result posted (*n* = 4), and no NSCLC (*n* = 5). Finally, 11 published articles in total were selected in our study up to May 2022.

### 3.2. Characteristics of the Studies Included

As shown in Table 1, eleven published articles containing 7,893 patients were included in the study. Between the 11 studies, 3, 2, 2, 2, 2 studies were performed in Asia, Worldwide, Europe, America, and Oceania, respectively. Most patients were treated with PPI before and/or shortly after the beginning of ICIs, and the type of ICI treatment was dominated by PD-(L) 1. In the Table 2, the 11 studies included were retrospective studies with result information of OS and/or PFS. The HR values were extracted from the univariate analysis of 5 studies and multivariate analysis of another 5 studies. The NOS score of all the included studies was greater than or equal to 6, which could be considered as high-quality articles.

### 3.3. The Association between PPI Use and OS

**Figure 2:**
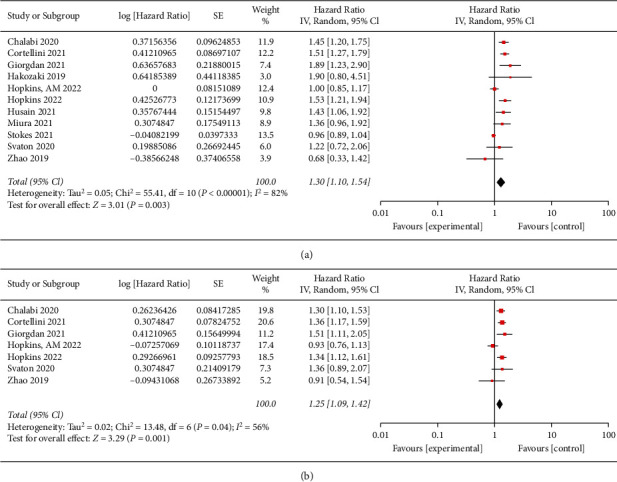
The forest plots of the hazard ratios (HRs) and 95% CIs for overall survival (a) and progression-free survival (b).

As indicated in Figure 2(a), 11 studies with 7,893 NSCLC patients were selected to perform meta-analysis for OS. The result revealed that in patients who had received ICIs treatment, the use of PPI was found to be significantly associated with poor OS (HR: 1.30, 95% CI: 1.10–1.54, *P*=0.003). Nevertheless, significant heterogeneity existed in this analysis (*I*^2^ = 82%, *P* < 0.001).

### 3.4. The Association between PPI Use and PFS

As shown in Figure 2(b), 7 studies with 3,454 NSCLC patients were selected to perform meta-analysis for PFS. The results revealed that PPI use was significantly associated with poor PFS in the patients who had received ICIs treatment (HR: 1.25, 95% CI: 1.09–1.42, *P*=0.001), with significant heterogeneity (*I*^2^ = 56%, *P*=0.04).

### 3.5. Subgroup Analysis of OS

To further assess the influence of PPI use in OS, the subgroup analyses were performed with regard to region, age, sample size, immunotherapy drugs, and duration of PPI exposure. As illustrated in Table 3, in terms of the region subgroup, PPI use was significantly related to poor OS in patients from Europe (HR = 1.48 [1.26, 1.74], *P* < 0.001) and worldwide multi-center studies (HR = 1.54 [1.24, 1.91], *P* < 0.001). The PPI use in patients with age less than or equal to 65, was found to be significantly associated to poor OS in the subgroup analysis related to age (HR = 1.56 [1.14, 2.15], *P*=0.006). With regard to the subgroup of sample size, the usage of PPI was found to be significantly associated to poor OS in studies with sample sizes less than or equal to 300 (HR = 1.37 [1.02, 1.84], *P*=0.04), and more than 300 (HR = 1.27 [1.04, 1.56], *P*=0.02). In the analysis of the subgroup of immunotherapy drugs, the PPI use was found to be significantly related to poor OS in patients who had received PD-1 treatment (HR = 1.37 [1.04, 1.79], *P*=0.03). With regard to the duration of PPI exposure subgroup, the result indicated that PPI use was significantly related to poor OS in patients who had received PPI treatment at 30 days before ICIs initiation (−30: HR = 1.89 [1.29, 2.78], *P*=0.001), and 30 days before and after starting ICIs treatment (±30: HR = 1.44 [1.27, 1.64], *P* < 0.001).

### 3.6. Subgroup Analysis of PFS

As in the subgroup analysis for OS, the PFS subgroup analyses were also performed with respect to region, age, sample size, immunotherapy drugs, and duration of PPI exposure, as shown in Table 4. In term of region subgroup, the use of PPI was significantly related to poor PFS in patients from Europe (HR = 1.36 [1.18, 1.57], *P* < 0.001) and worldwide multi-center studies (HR = 1.34 [1.16, 1.55], *P* < 0.001). The PPI usage indicated significant association with poor PFS in patients having age above 65 years (HR = 1.36 [1.18, 1.57], *P* < 0.001) in the age subgroup. In case of the sample size subgroup, the PPI use was found to be significantly related to poor PFS in studies with the sample size more than 300 (HR = 1.23 [1.04, 1.44], *P*=0.01). With regard to the subgroup analysis of immunotherapy drugs, PPI use was found to be significantly related to poor PFS in patients having received PD-L1 treatment (HR = 1.33 [1.19, 1.49], *P* < 0.001). In case of subgroup regarding duration of PPI exposure, the result revealed that PPI use was significantly associated with poor PFS in patients treated with PPI at 30 days before starting the ICIs treatment (−30: HR = 1.51 [1.11, 2.05], *P*=0.008), and 30 days before and after starting the ICIs treatment (±30: HR = 1.32 [1.20, 1.45], *P* < 0.001).

### 3.7. Publication Bias

**Figure 3:**
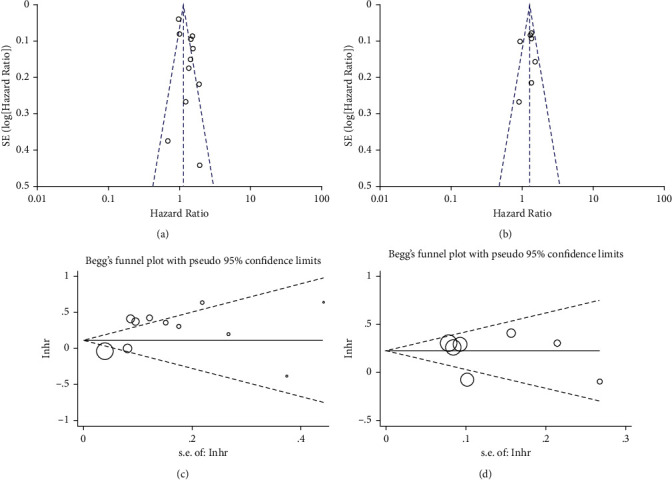
The Publication bias. (a) Funnel plot analysis of overall survival (OS). (b) Funnel plot analysis of progression-free survival (PFS). (c) Begg's funnel plots for evaluating the publication bias of overall survival (OS). (d) Begg's funnel plots for evaluating the publication bias of progression-free survival (PFS).

The funnel plots assisted in assessing the publication bias, while the results revealed that there was no significant asymmetry about HR (OS or PFS), confirming that there was little possibility of publication bias (Figures 3(a) and 3(b)). In addition, Begg's tests and Egger's tests were also used to verify whether there is publication bias. As shown in Figures 3(c) and 3(d), there was no significant publication bias in the HR value of OS (Begg's test, *P*=0.640; Egger's test, *P*=0.059) and PFS (Begg's test, *P*=0.368; Egger's test, *P*=0.724).

### 3.8. Sensitivity Analysis

**Figure 4:**
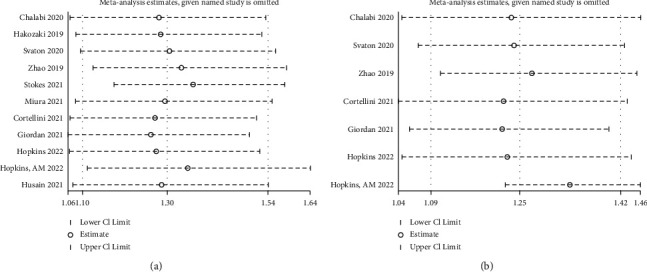
The sensitivity analysis. (a) Sensitivity analysis for hazard ratio (HR) of overall survival (OS). (b) Sensitivity analysis for hazard ratio (HR) of progression-free survival (PFS).

We performed sensitivity analysis on the included literature. It is obvious from the results that no single study had a great impact on the final combined HR value of OS and PFS. Therefore, we believe that the combined results of this study were reliable and robust (Figures 4(a) and 4(b)).

## 4. Discussion

The clinical efficacy or survival outcome of the use of PPI combined with ICIs in patients with NSCLC has not been known properly. Nevertheless, the following points deserve our attention. First, PPI has been proved to play a pivotal role in operating the immunologic defense to the treatment of tumors by regulating the activity or compositions of the gastrointestinal bacteria [18]. Second, for patients with gastric ulcers or gastrointestinal bleeding history, PPI could be prophylactically used to prevent the occurrence of stress ulcers [10]. Third, since PPI could greatly increase the sensitivity of cancer patients to chemotherapy, it was often used together with other types of anticancer drugs, such as ICIs, nonetheless, the clinical efficacy of the combination has remained unknown until today. Finally, whether the combination of PPI and ICIs would increase the adverse reactions related to the two drugs. For example, the long-term use of omeprazole would correspondingly increase the risk of liver failure and chronic kidney disease, thus affecting the efficacy of cancer treatment. A study revealed that the PPI use would significantly impact the composition of the gastrointestinal bacteria and greatly reduce the clinical efficacy of ICIs [19, 20]. In the study of Derosa et al. [21], it is shown that for advanced renal cell carcinoma and NSCLC patients, the use of antibiotics combined with ICIs would also lead to poor OS and PFS, which may be due to the fact that antibiotics greatly inhibit the diversity and abundance of intestinal flora, leading to the inability to fully mobilize the immune function. This may be similar to the reason why PPI combined with ICIs produced poor clinical efficacy. Other studies revealed that the PPI use would not have any influence on the clinical efficacy of ICIs [22]. Hence, to determine the correlation between the clinical efficacy of ICIs in NSCLC and the use of PPI, a meta-analysis was conducted.

On the one hand, compared with the study of Wei et al., Li et al., Qin et al., and Sophia et al. [10, 15, 23, 24], this study included more studies on NSCLC (*n* = 11 vs. *n* = 6 for Wei et al., *n* = 7 for Li et al., *n* = 7 for Qin et al., *n* = 4 for Sophia et al.) and more patients (*n* = 7,893 vs. *n* = 5,114 for Wei et al., *n* = 1,428 for Li et al., *n* = 3,647 for Qin et al., *n* = 2,940 for Sophia et al.), especially including two articles published in 2022. On the other hand, based on more factors, like the duration of exposure to PPI, the PPI treatment, type of ICIs treatment, sample size, age, and region, the subgroup analysis was conducted. It would further help to understand the actual role of PPI in the combined use of ICIs drugs in cancer treatment. Hence, it is believed that our study has been very necessary and could provide certain basis for the rationale in the usage of PPI in clinical practices.

In patients treated with ICIs, the result indicated that PPI use was associated significantly to poor PFS (HR: 1.25 [1.09–1.42]) and poor OS (HR: 1.30 [1.10–1.54]). Nonetheless, in patients who had received ICIs treatment, the PPI use was not found to be associated with PFS and OS according to the study of Li et al. and Meng et al. [15, 25] in 2020. This could be due to the fact that the PPI use would lead to greater changes in the activity and composition of the gastrointestinal microbiota, which would be related to the tolerance of the T-cells. Simultaneously, the PPI use would not only affect the microbiota of gastrointestinal tract, but also would have a certain impact on the growth, metastasis, and progression of the tumor. In the study of De Milito et al. and Bellone et al. [26, 27], it was proposed that PPI could impact the tumor growth and metastasis by regulating the acidic microenvironment of the tissues around tumors. Meanwhile, the use of PPI severely inhibited the hydrogen ion ATPase pump, thus reversing the pH gradient of acidic microenvironment [27]. Besides, the use of PPI greatly promoted the generation of M2-subtype macrophages and pro-inflammatory cytokines (such as interleukin 7) [28]. All of these will reduce the immunosuppressive ability of tumor microenvironment and greatly inhibit the activity of ICIs [29]. Moreover, PPI would also increase the sensitivity of patients to chemotherapy and immunotherapy [9]. Hence, based on the PPI use, predicting the clinical efficacy of ICIs in NSCLC patients is highly difficult. Besides, further basic and relevant clinical studies would be required.

On factors like duration of PPI exposure, type of ICIs treatment, sample size, age, and region, a subgroup analysis was conducted to explore further the correlation between the clinical efficacy of ICIs and the use of PPI. PPI use was found to be significantly related to poor OS in case of the analysis on the region subgroup (Europe: HR = 1.48 [1.26, 1.74], Worldwide: HR = 1.54 [1.24, 1.91]) and poor PFS (Europe: HR = 1.36 [1.18, 1.57], Worldwide: HR = 1.34 [1.16, 1.55]) in patients from Europe and worldwide multi-center studies. It was suggested that the multi-center research projects need to be promoted between regions and countries. In term of sample size subgroup, PPI usage was found to be significantly related to poor OS in studies with the sample size less than or equal to 300 (HR = 1.37 [1.02, 1.84]), and more than 300 (HR = 1.27 [1.04, 1.56]), and poor PFS in studies with the sample size more than 300 (HR = 1.23 [1.04, 1.44]). In clarifying the correlation between clinical efficacy of ICIs and the PPI usage, the sample size played a crucial role, as confirmed from the results, and it has been recommended to include as many relevant samples as possible in clinical studies. For age subgroup, PPI use was significantly related to poor OS in patients with age less than or equal to 65 years (HR = 1.56 [1.14, 2.15]), and to poor PFS in patients with age more than 65 years (HR = 1.36 [1.18, 1.57]). People of different ages have different sensitivities to PPI. Hence, the clinical use of PPI needs to be cautious. With regard to the duration of PPI exposure subgroup, the PPI use was found to be significantly related to poor OS (−30: HR = 1.89 [1.29, 2.78], ±30: HR = 1.44 [1.27, 1.64]) and poor PFS (−30: HR = 1.51 [1.11, 2.05], ±30: HR = 1.32 [1.20, 1.45]) in patients treated with PPI drugs at 30 days before and/or after starting ICIs treatment. In the clinic, we need to stop the application of PPI immediately before and/or after starting the ICIs treatment, so as to provide the patients with good therapeutic effect. Finally, for type of ICIs treatment subgroup, PPI use displayed a poor prognosis for patients having received PD-L1 or PD-1 treatment, which possibly was related to the limited sample size included in this study. In addition, based on the above discussion, we suggest that when PPI is used in combination with ICIs in clinical practice, appropriate adjustment of the dysbiosis of organism and gastrointestinal bacterial colony disorder caused by the use of PPI may greatly improve the clinical efficacy and prognosis of relevant patients. Hence, to clarify the relationship between the clinical efficacy of ICIs in NSCLC and the PPI usage, more relevant research would be needed.

Our study had certain limitation. First, some PFS data were missing in the included studies, and this study only extracted the HR value and 95% CI value of the included study rather than the initial data of the study, which could have a greater impact on our results. Second, the studies included were retrospective studies. In the process of extracting data, such as sample size, type of ICIs treatment, and duration of PPI exposure, region, and age, the detailed information of relevant data could not be known, which could lead to certain limitations in the overall and subgroup analysis of this study. Third, this study only included studies published in English, while those published in other languages, for instance, Chinese, were not included, which could indirectly lead to increased heterogeneity of this study. Finally, it was found that there was no direct study proving the association between the PPI use and the clinical efficacy of ICIs in NSCLC. Hence, more research related to this becomes imperative.

## 5. Conclusion

In conclusion, our meta-analysis found that PPI combined with ICIs in the treatment of NSCLC patients possibly resulted in poor OS and PFS. In term of subgroup analysis, PPI use had a poor prognosis for patients having received PD-L1 or PD-1 treatment, or those who received PPI drugs at 30 days before and/or after ICIs initiation. Simultaneously, the effect of PPI on patients of different age groups was also different. Hence, in clinical practice, we need to be extremely cautious in the PPI use to avoid the influence in the efficacy of ICIs. Nevertheless, the concrete mechanism between the use of PPI and the efficacy of ICIs needs to be further studied, so as to further improve the clinical treatment level of the related tumors.

## Figures and Tables

**Table 1:** Baseline characteristics of the included studies.

Author	Year	Age	Region	Cancer type	ICI treatment	PPI treatment	No. of PPI	Patients	PPI exposure
Chalabi et al. [30]	2020	NA	Worldwide	NSCLC	PD-L1	Omeprazole, pantoprazole, lansoprazole, rabeprazole, esomeprazole, dexlansoprazole	234	757	Prior, within (30 days)
Hakozaki et al. [31]	2019	67	Asia	NSCLC	PD-1	NA	47	90	Prior (30 days)
Svaton et al. [32]	2020	67	Europe	NSCLC	PD-1	Omeprazole, pantoprazole, lansoprazole	64	224	Prior, within (30 days)
Zhao et al. [33]	2019	62	Asia	NSCLC	PD-1, other	NA	40	109	Prior, within (30 days)
Stokes et al. [34]	2021	69	America	NSCLC	PD-(L)1	Omeprazole (majority)	2159	3634	Within (90 days)
Miura et al. [35]	2021	65	Asia	NSCLC	PD-1	Lansoprazole, rabeprazole, Esomeprazole	163	300	Within
Cortellini et al. [36]	2021	70.1	Europe	NSCLC	PD-L1	NA	474	950	Prior, within (30 days)
Giordan et al. [29]	2021	63.9	Worldwide	NSCLC	PD-(L)1	Pantoprazole, esomeprazole, lansoprazole, Rabeprazole, omeprazole	47	212	Prior (30 days)
Hopkins et al. [37]	2022	NA	Oceania	NSCLC	PD-(L)1	NA	441	1202	Prior, within (30 days)
Hopkins et al. [38]	2022	NA	Oceania	NSCLC	PD-L1	Omeprazole,pantoprazole, esomeprazole, lansoprazole, rabeprazole, dexlansoprazole, vanoprazan	1225	4458	Within
Husain et al. [39]	2021	NA	America	NSCLC	PD-(L)1	NA	149	415	Within

NSCLC, non-small cell lung cancer; PPI, proton pump inhibitor; PD-1, programmed cell death protein-1; PD-L1, programmed cell death ligand 1; NA, not available; ICI, immune checkpoint inhibitor.

**Table 2:** Quality assessment and prognostic information of the included studies.

Author	Year	Method	Outcome	HR (95% CI) for OS	HR (95% CI) for PFS	Analysis	NOS score
Chalabi et al. [30]	2020	RE	OS/PFS	1.45 (1.20–1.75)	1.30 (1.10–1.53)	NA	8
Hakozaki et al. [31]	2019	RE	OS	1.90 (0.80–4.51)	NA	M	6
Svaton et al. [32]	2020	RE	OS/PFS	1.22 (0.72–2.05)	1.36 (0.89–2.06)	M	8
Zhao et al. [33]	2019	RE	OS/PFS	0.68 (0.33–1.43)	0.91 (0.54–1.54)	U	8
Stokes et al. [34]	2021	RE	OS	0.96 (0.89–1.04)	NA	M	7
Miura et al. [35]	2021	RE	OS	1.36 (0.96–1.91)	NA	M	7
Cortellini et al. [36]	2021	RE	OS/PFS	1.51 (1.28–1.80)	1.36 (1.17–1.59)	U	8
Giordan et al. [29]	2021	RE	OS/PFS	1.89 (1.23–2.90)	1.51 (1.11–2.05)	M	7
Hopkins et al. [37]	2022	RE	OS/PFS	1.53 (1.21–1.95)	1.34 (1.12–1.61)	U	7
Hopkins et al. [38]	2022	RE	OS/PFS	1.00 (0.85–1.17)	0.93 (0.76–1.13)	U	8
Husain et al. [39]	2021	RE	OS	1.43 (1.06–1.92)	NA	U	6

OS, overall survival; PFS, progression-free survival; HR, hazard ratio, NA, not available; U, univariate; M, multivariate; NOS, Newcastle-Ottawa Scale; RE, retrospective.

**Table 3:** The subgroup analysis of the correlation between the use of PPI and clinical efficacy of ICIs for overall survival.

Subgroup	No. of studies	OS hazard ratios (95% CI)	*P*value	Heterogeneity	
				*I* ^2^ (%)	*P*value
Region					
Worldwide	2	1.54 [1.24, 1.91]	<0.001	19.00	0.27
Asia	3	1.21 [0.74, 1.98]	0.44	47.00	0.15
Europe	2	1.48 [1.26, 1.74]	<0.001	0	0.45
America	2	1.14 [0.77, 1.68]	0.51	85.00	0.01
Oceania	2	1.23 [0.81, 1.86]	0.34	88.00	0.004

Age					
≤65	3	1.56 [1.14, 2.15]	0.006	27.00	0.24
>65	4	1.26 [0.89, 1.79]	0.19	88.00	<0.001

Sample size					
≤300	5	1.37 [1.02, 1.84]	0.04	37.00	0.17
>300	6	1.27 [1.04, 1.56]	0.02	89.00	<0.001

Immunotherapy drug					
PD-L1	3	1.30 [0.99, 1.69]	0.06	86.00	<0.001
PD-1	3	1.37 [1.04, 1.79]	0.03	0	0.69
PD-1, other	1	0.68 [0.33, 1.42]	0.3	NA	NA
PD-(L)1	4	1.37 [0.98, 1.92]	0.07	88.00	<0.001

PPI exposure					
−30	2	1.89 [1.29, 2.78]	0.001	0	0.99
±30	5	1.44 [1.27, 1.64]	<0.001	19.00	0.3
∞	4	1.10 [0.93, 1.30]	0.27	69.00	0.02

OS, overall survival; PD-1, programmed cell death protein-1; PD-L1, programmed cell death ligand 1; HR, hazard ratio; NA, not available; PPI: proton pump inhibitors.

**Table 4:** The subgroup analysis of the correlation between the use of PPI and clinical efficacy of ICIs for progression-free survival.

Subgroup	No. of studies	PFS hazard ratios (95% CI)	*P* value	Heterogeneity	
				*I* ^2^ (%)	*P* value
Region					
Worldwide	2	1.34 [1.16, 1.55]	<0.001	0	0.4
Asia	1	0.91 [0.54, 1.54]	0.72	NA	NA
Europe	2	1.36 [1.18, 1.57]	<0.001	0	0.99
Oceania	2	1.12 [0.78, 1.60]	0.54	86.00	0.008

Age					
≤65	2	1.23 [0.75, 2.00]	0.41	63.00	0.1
>65	2	1.36 [1.18, 1.57]	<0.001	0	0.99

Sample size					
≤300	3	1.31 [1.00, 1.71]	0.05	25.00	0.26
>300	4	1.23 [1.04, 1.44]	0.01	71.00	0.01

Immunotherapy drug					
PD-L1	2	1.33 [1.19, 1.49]	<0.001	0	0.69
PD-1	1	1.36 [0.89, 2.07]	0.15	NA	NA
PD-1, other	1	0.91 [0.54, 1.54]	0.72	NA	NA
PD-(L)1	3	1.17 [0.73, 1.88]	0.52	85.00	0.009

PPI exposure					
−30	1	1.51 [1.11, 2.05]	0.008	NA	NA
±30	5	1.32 [1.20, 1.45]	<0.001	0	0.71
∞	1	0.93 [0.76, 1.13]	0.47	NA	NA

PFS, progression-free survival; PD-1, programmed cell death protein-1; PD-L1, programmed cell death ligand 1; HR, hazard ratio; NA, not available.

## Data Availability

All data generated or analyzed in order to support the findings of this study are included within the article.
